# The Work of the American Red Cross Bureau of Tuberculosis in France

**Published:** 1919-01

**Authors:** 


***The Work of the American Red Cross Bureau of Tuberculosis in France.*** By WILLIAM CHARLES WHITE, Chief of Bureau. *The American Journal of Medical Sciences*, September, 1918.

In the third year of the war the Rockefeller Foundation planned to undertake, in co-operation with the French, an anti-tuberculosis campaign in France. After America joined the war, the American Red Cross announced its intention to contribute to this work, and the Bureau of Tuberculosis was founded. The Rockefeller Commission undertook the study of the existing conditions, the provision of dispensary service, and an educational campaign, while the Red Cross took over the provision of hospitals, improvement in the condition of temporary hospitals, assistance to French organizations, etc.

The work of the Bureau of Tuberculosis may be summarized as follows:

Provisions of new beds in existing French institutions, Bligny and St. Joseph.

Improvement of conditions in existing French institutions.

The Bureau of Tuberculosis undertook to assist the French institutions in the care of completely discharged soldiers who had been drafted from the devastated regions, rapatriés, both French and foreign refugees, and the civilian population. The seven sets of barracks constructed by the Assistance Publique for tuberculous patients were provided with new kitchens, recreation rooms, cure halls, etc. and the number of patients soon increased from 174 to

657. Additional nurses were also provided by the A. R. C. A new division for institutions, dealing with tuberculosis outside of Paris, was created, and France was visited department by department. The following types of institutions for tuberculous patients were found :

1. Hôpitaux Sanitaires.
2. Stations Sanitaires.
3. Hôpitaux mixtes, taking military and civil cases.
4. Hôpitaux complémentaires (annexes to military hospitals.)
5. Sanatoria.
6. Hospitals for bone and gland tuberculosis.

These provided a total of 11,000 beds for tuberculosis, for a population of 39,500,000 people, with a total death rate from tuberculosis of 84,443 in 1913. Many of the existing institutions required additional food, bedding, and equipment, which the A. R. C. undertook to furnish at an approximate cost of 100,000 frcs. per month. Within five months' time, assistance was given to about 12,000 tuberculous patients.

The Bureau of Tuberculosis financed the Hopital d'Ormesson, thus continuing the use of 160 beds for tuberculous soldiers, which would otherwise have been unavailable, and contributed to the opening of a hospital of 40 beds for tuberculous women. Its work in that direction continues, giving excellent results.

*Provision of New Hospitals for the Civilian Population.*

France had been able to do but very little for the tuberculous rapatriés, refugees and civilians. The Bureau of Tuberculosis undertook to assist in this most urgent need, and chose for its field of operation the two largest centers of population in France, Paris and Lyon. Two properties were placed at the disposal of the A.R. C. by the Department of the Seine. About 150 women and children are cared for at present. The future plans contemplate the housing of about 2000 tuberculous persons, grouped as follows : a sanatorium for women, a detention house for entering children, a hospital for tuberculous children, a precéntorium for children of tuberculous parents, and a colony for families with a tuberculous member from whom they cannot be separated. The work is being carried out in co-operation with the Department of the Interior of the French Government, the dispensary service of the Rockefeller Foundation, and the Hospital Admission Bureau.

The plan of the home colony contemplates placing families of refugees with a tuberculous member in small houses for the duration of the war. Each house consists of two sleeping rooms and a living room, with a porch for the consumptive member. Each family will perform its household duties, the children will attend open-air schools, the members able to work will be given educational training, and a careful instruction in hygienic living.

Lyon, situated on the Evian line through which the rapatriés enter France, forms a sort of clearing station for tuberculous cases. The Bureau of Tuberculosis received, in that town, a large hospital building and five barraks for the housing of rapatriés, women and children, suffering from tuberculosis. The Red Cross provides the medical care, most of the nursing, and pays a rate of between 3 and 6 frcs. per day, per patient, for food, clothing and other expenses. The activity of the Bureau is to be greatly increased, so as to provide for the local civilian population which, so far, is not permitted to enter the Asile Ste. Eugenie.

*Co-operation with the Rockefeller Commission.*

The Bureau of Tuberculosis accepted the responsibility of providing all the additional equipment necessary for the work of the Rockefeller Foundation in the 19th arrondissement of Paris, and in its model Department of Eure-et-Loire. This assistance consisted of appropriations for dispensary construction, appropriations for educational pamphlets, and assistance in the provision of hospital beds.

*The Hospital Admission Bureau.*

The Hospital Admission Bureau, as part of the Bureau of Tuberculosis, deals with all individual cases of tuberculosis requesting hospital assistance. Such cases are recommended by the Admission Bureau to the existing French hospitals for tuberculous patients, and accepted by the latter upon payment by the Bureau of a per capita cost, ranging from 4.5 to 6.5 frcs. per day. From December 25, 1917, to April 1918, 750 cases of tuberculosis were thus provided for. This division of the Bureau, besides giving assistance to a number of patients, established the beginning of a central registration of living tuberculous cases which had not, up to then, existed in France.

*Provision of Hospital for American Soldiers.*

In spite of an extremely careful exclusion of tuberculosis cases in the draft, the American Army has to deal with a number of tuberculous soldiers for whom the Red Cross is undertaking the provision of a hospital near Bordeaux. A similar institution will, if necessary, be provided for the hospital at Savenay, where, up to the present, tuberculous cases have been cared for in tents.

*The Problem of the Serbians in France.*

The Bureau of Tuberculosis started a division for the study of the question of assistance to the tuberculous Serbian refugees in

France. Serbian camps were visited and arrangements were made for the establishment of a hospital for Serbians with tuberculosis, which, it is hoped, will be under way by the end of April.

*Departmental Reorganization.*

The department of Indre-et-Loire, where a hospital for tuberculosis was founded at Tours, at the request of the French " Secours aux Blessés Militaires ", may be taken as a typical example of the methods pursued. The Red Cross agreed to give 250,000 frcs. towards the hospital, with the understanding that the new hospital committee would take steps to organize the existing dispensaries under its own supervision, that it would create new dispensaries in the department, employ salaried, trained, visiting nurses for the new dispensaries and for supervision of the visiting work in the communes, and that plans would be made for the early provision of hospital beds for urgent cases. The Red Cross pledged itself to assist in this work, and in the other needs of the department as they arose.

In March 1918, a new division of the Bureau of Tuberculosis was created, which undertook to visit France department by department, with a view to applying the above described methods throughout the country. The visiting work, carried out in correlation with the Rockefeller Foundation, began in the departments of Loire-Inférieure, Ille-et-Vilaine, Côtes-du-Nord and Finistère. This work will probably consume over a year, but it will leave a lasting impression of the American effort in France, and place the control of tuberculosis on a firm basis.

